# Hypocalcemia in cancer patients: An exploratory study

**DOI:** 10.1097/j.pbj.0000000000000045

**Published:** 2019-07-08

**Authors:** José António Ferraz Gonçalves, Telma Costa, Joana Rema, Cristina Pinto, Miguel Magalhães, Ana Esperança, Luciana Sousa

**Affiliations:** a Instituto Português de Oncologia, Porto, Portugal; b Centro Hospitalar de S. João, Porto, Portugal; c Hospital de Faro, Faro, Portugal; d USF Barão de Nova Sintra, Porto, Portugal; e USF Mais Saúde, Ponte de Lima, Portugal; f Hospital de Braga, Braga, Portugal.

**Keywords:** cancer, hypocalcemia, prognosis

## Abstract

**Introduction:**

Calcium has several physiological functions and when it registers abnormal serum levels those functions may be impacted with potentially severe consequences. There is much research on hypercalcemia in cancer patients, but there are no recent studies on the prevalence of hypocalcemia in those patients. However, there has been an evolution in oncology, namely, new drugs that may directly or indirectly result in hypocalcemia. The primary aim was to explore the association of hypocalcemia with the diverse types of cancer. The secondary aim was to study the influence that hypocalcemia might have on survival.

**Methods:**

Review of the records of patients ≥18 years old, with total calcium <2.0 mmol/L measured in 2013 in a cancer center.

**Results:**

Eight hundred twenty-nine patients were included. Four hundred thirty-nine (53%) were male. The median age was 63 years. The most frequent cancer diagnoses were hematological 196 (24%) and colorectal 111 (13%). Six hundred thirty-eight patients had measured serum albumin, with a median of 25.5 g/L (14–47). When corrected for albumin level, calcium was in the normal range in 210 (33%) cases. The median survival of patients with corrected calcium lower than normal was 479 days (95% confidence interval [CI]: 309–649) and that of patients with normal corrected calcium was 62 days (95% CI: 33–91), *P* < .001. In a multivariate analysis, age, primary cancer, and albumin were significantly associated with survival.

**Conclusion:**

Hypocalcemia is associated with several types of cancer. A low calcium level is not by itself a factor of a poor prognosis since other factors seem to be more important.

## Introduction

Serum calcium exists in 3 forms: free or ionized calcium accounting for 50% of total serum calcium; calcium bound to plasma proteins (80% bound to albumin), accounting for about 40% of total serum calcium; and calcium in compounds such as bicarbonate, lactate, phosphate, and citrate.^1^ Ionized calcium is the physiologically active form. The level of total serum calcium is influenced by the serum proteins, mainly albumin. That level may be outside normal limits, maintaining a normal level in the physiologically active form.

Hypercalcemia is the object of extensive study in cancer patients,^2–4^ but hypocalcemia is not reported as often. However, hypocalcemia can be as serious as hypercalcemia. Patients with mild hypocalcemia may be asymptomatic, but when symptomatic, unspecific manifestations such as fatigue, irritability, anxiety, and depression may occur. However, the characteristic manifestations result in muscular irritability with tetany (which can be proven by the Chvostec and Trousseau signs), perioral numbness, distal paresthesias and muscle cramps. If severe, hypocalcemia may cause bronchospasm and/or laryngospasm, seizures, and at the cardiac level, hypocalcemia may prolong QT and ST intervals in the electrocardiogram, and cause a 2:1 block.^5^

The occurrence of hypocalcemia in cancer patients may have many causes.^6^ Drugs commonly used^1^: several chemotherapeutic agents are implicated in cases of hypocalcemia^7,8^; bisphosphonates frequently used to treat cancer patients with bone metastases or cancer types with a high risk of bone involvement may also cause hypocalcemia^9,10^; proton pump inhibitors, widely used, may decrease calcium absorption and may cause hypocalcemia directly,^11^ through hypomagnesemia^12^ or both. Tumor lysis syndrome, resulting from the rapid death of tumor cells, is particularly common in patients with hematological malignancies with rapid cellular turnover, but it also occurs in solid tumors,^13,14^ and is a potentially deadly complication of tumors or their treatment as it causes acute renal failure and multiple metabolic alterations including hypocalcemia. Osteoblastic bone metastases may cause hypocalcemia by avidly capturing calcium—hungry bone syndrome—a situation more commonly associated with prostate cancer,^15,16^ although it may also be associated with other tumors.^6^ Other causes of hypocalcemia are hypoparathyroidism secondary to thyroid surgery or radiotherapy for the treatment of head and neck cancer, infections or renal failure,^6^ and malnutrition.

As far as we could find from reviewing the relevant literature, there are only 2 studies on the prevalence of hypocalcemia in cancer patients,^6,17^ and the most recent one was published in 1991.^17^ A recently published review confirms this finding.^18^ As there have been evolutions in oncology, such as the improvement in survival and the introduction of drugs that may directly or indirectly result in hypocalcemia, we thought that a new review would be appropriate, and the authors cited above also have the same opinion.^18^ For those reasons, we carried out this study on hypocalcemia associated with malignant diseases.

The primary aim was to explore the association of hypocalcemia with different types of cancer. The secondary aim was to study the influence that hypocalcemia might have on survival.

## Methods

This study was carried out in a comprehensive oncology center which admits about 10,000 new patients per year. For this retrospective study, all records of blood tests with total calcium below the lower limit of normal (2.20 mmol/L) in 2013 were collected. Many were marginally low, unlikely to be clinically significant. Therefore, only the records with a total calcium <2.0 mmol/L were included. This study was limited to patients ≥18 years old. A total of 829 patients meet the inclusion criteria.

Whenever an albumin level was available, which occurred in 638 cases (77%), total calcium was corrected for albumin level according to the formula: corrected calcium (mmol/L) = measured calcium (mmol/L) + 0.02 × (40 − albumin [g/L]).

Descriptive statistics methods were used for analyzing the data. To evaluate the existence or not of associations between variables, the chi-squared or the Mann–Whitney tests were used, as appropriate. Survival curves were calculated using Kaplan–Meier estimator and compared using the log-rank test. For multivariate analysis, the Cox regression was used. The level of significance was deemed to be 0.05. Missing data, which occurred in patients without an available albumin level (n = 191, 23%) were omitted.

This study was approved by the Ethics Committee of the Hospital.

## Results

The median age was 63 years (18–93) and 439 (53%) were male. The most frequent cancer diagnoses were hematological, 195 (24%), followed by colorectal, 111 (13%), and lung, 86 (10%), cancers (Table 1).

**Table 1:**
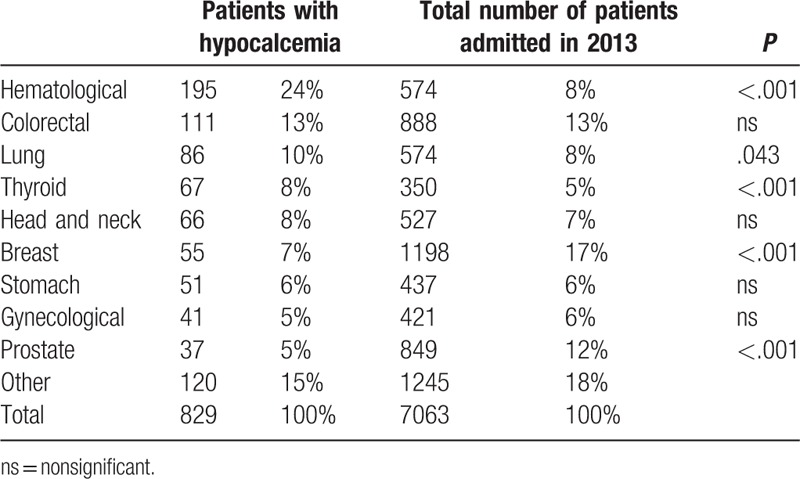
Cancer type

The most common hematological malignancy was multiple myeloma followed by non-Hodgkin lymphoma (Table 2). Compared to the total number of patients admitted to the hospital in 2013, those with hypocalcemia presented a higher proportion of hematological malignancies, lung cancer and thyroid cancer, and a lower proportion of breast and prostate cancers (Table 1).

**Table 2:**
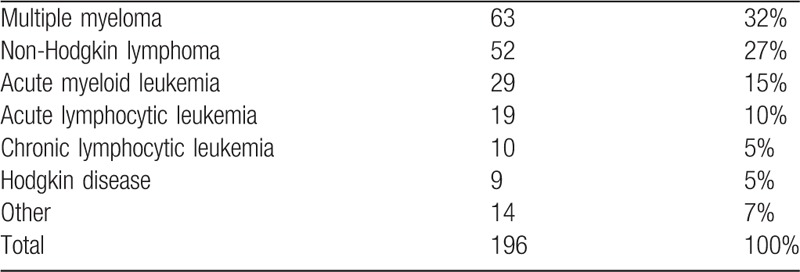
Hematological malignancies

Six hundred thirty-eight patients had measured their serum albumin, with a median of 25.5 g/L (14–47). When corrected for albumin level, the calcium was in the normal range in 210 (33%) cases and remained low in 428 (67%). The median albumin level was 28 g/L (14–47) in patients with the low corrected calcium and 23 g/L (14–29) in those with normal corrected calcium (*P* < .001). The distribution of corrected calcium by the quartiles of albumin also showed that normal corrected calcium concentrated in the lower quartiles (Table 3).

**Table 3:**
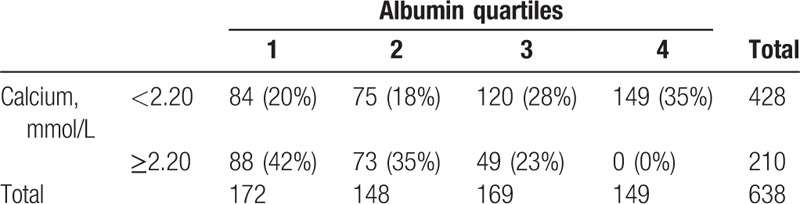
Distribution of patients with corrected calcium by albumin quartiles

The 2-year survival rate of the 428 patients with a low corrected calcium was 44% (189 patients), whereas the 2-year survival rate of the 210 patients with a normal corrected calcium was only 25% (53 patients) (*P* < .001). The median survival of patients with corrected calcium lower than normal was 479 days (95% confidence interval [CI]: 309–649) and that of patients with normal corrected calcium was only 62 days (95% CI: 33–91), *P* < .001.

In a univariate analysis age, sex, primary cancer type, albumin, and corrected calcium are shown to be significantly associated with survival (Table 4), but the measured calcium was not. In the multivariate analysis only age, the primary cancer, and albumin were significantly associated with survival (Table 5): patients older than 63 years old (the median age) had a lower survival relative to the younger ones; head and neck, colorectal, thyroid, and hematological cancers had a higher survival rate and lung cancer a lower rate compared with the reference category—stomach cancer. Survival increased as the quartile of albumin level also increased.

**Table 4:**
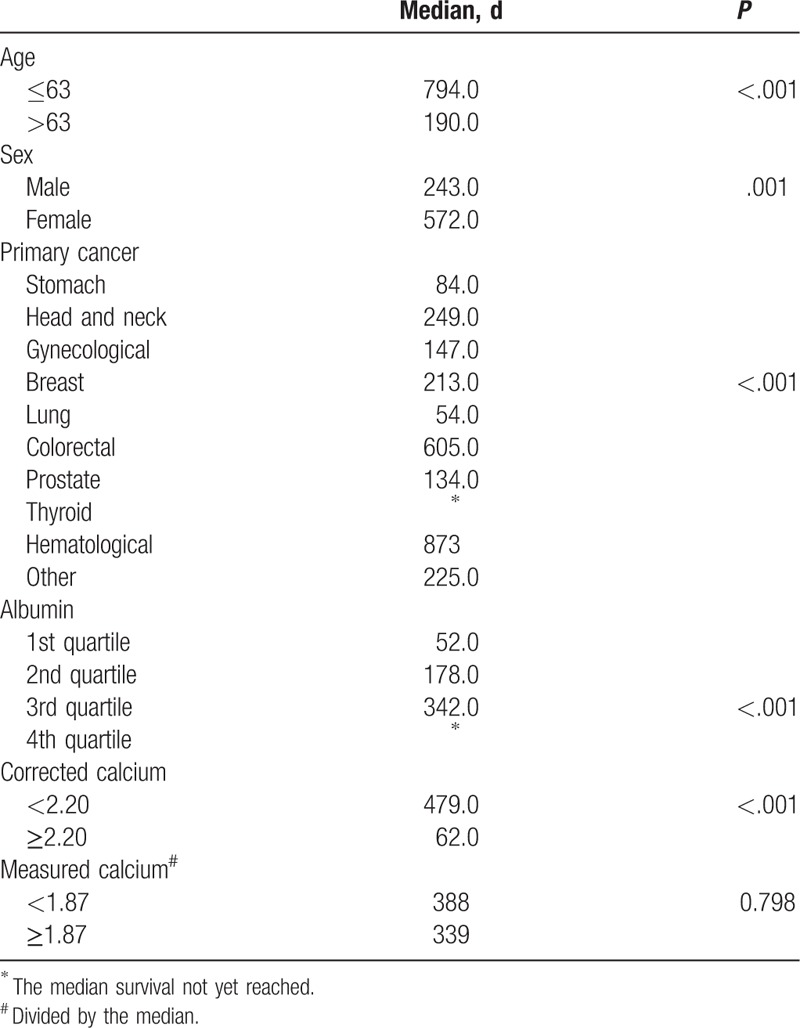
Univariate analysis of factors related to survival

**Table 5:**
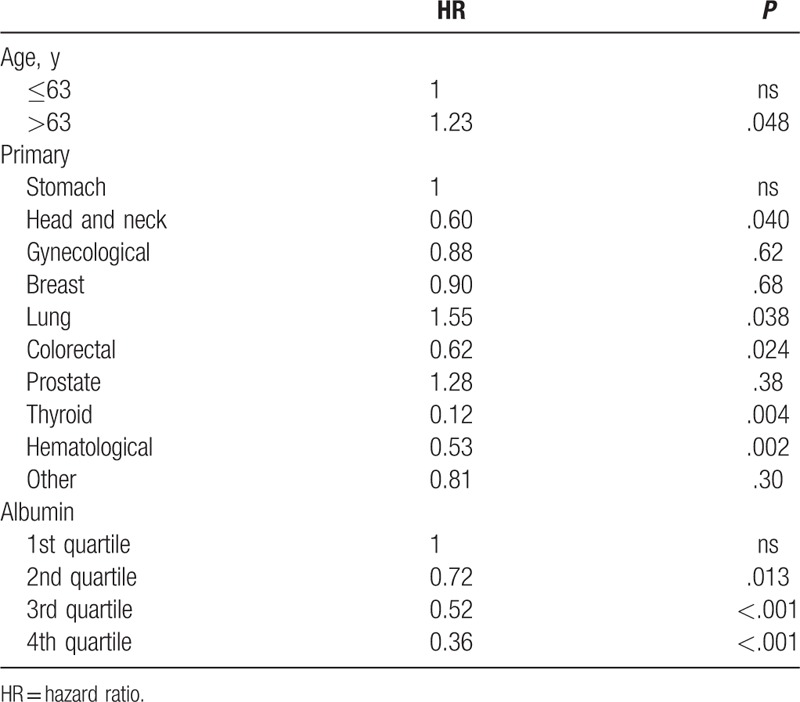
Multivariate analysis of factors related to survival

## Discussion

Hypocalcemia is a frequent occurrence in cancer patients. In this study, the most frequent diagnosis as a group was hematological malignancies. Multiple myeloma was the most frequent diagnosis among them. The osteolytic nature of bone involvement in multiple myeloma generally results in hypercalcemia, but its treatment with bisphosphonates, mainly zoledronate, but also pamidronate, and renal failure may result in hypocalcemia.^19,20^ The tumor burden of acute leukemia and lymphoma with rapid cell turnover and massive cell lysis due to treatments such as chemotherapy, radiotherapy or even dexamethasone may result in various metabolic alterations including hypocalcemia.^14,21^ Comparing the cases with hypocalcemia with the total number of patients admitted in 2013, to have a rough idea of the proportion of cases, some cancer types, such as hematological, lung, and thyroid, were found to have a higher proportion and others, such as breast and prostate, a lower proportion.

As a secondary aim, we looked at the influence that hypocalcemia could have on survival and the result surprised us. It would be expected that a normal corrected calcium level would be associated with better survival than a low corrected calcium level. However, an unexpected observation in this study was the highly significant difference in terms of survival of patients with low corrected calcium. As it was a counter-intuitive result, and since the corrected calcium depends on the level of albumin and measured calcium was not associated with survival, corrected calcium was excluded from the multivariate analysis. How can this unexpected result be explained? It should be noted that patients with normal corrected calcium had statistically very significant lower albumin level than patients with low corrected calcium and low albumin is a poor prognosis factor.^22^ This fact may explain the observed difference in survival. The cancer type and age also had a significant influence on survival, as expected. This study was not designed to explore the prognosis of these patients in depth, but it can be concluded that other factors are much more important than hypocalcemia where survival is concerned.

The surveys previously mentioned, that were published in 1986 and 1991, are very different in many aspects^6,17^: in the clinical setting, in the number of patients, in the neoplasms included, and in the formulas used to correct calcium for albumin. Therefore, it is very difficult to compare them. The comparison with the present study is also obviously very difficult, but trying to find some points for comparison we can note, for example, that: both previous studies included much less patients with hypocalcemia than our study; all studies concluded that in patients with malignant diseases hypocalcemia is a “fairly common finding”^6,17^; hematological neoplasms which were the most frequent in our study do not appear in the study of D’Erasmo et al^17^ but they were present in the study by Blomqvist et al,^6^ although in a much lower percentage and were only represented by non-Hodgkin lymphoma and Hodgkin disease. In the Blomqvist study, pancreatic cancer was the most frequent cancer type with a percentage of 22%, but this means 2 out of 9 cases. Both studies concluded that there were no evident specific symptoms attributable to hypocalcemia, as the symptoms of the primary disease probably obscured any others which could be present. The study by Blomqvist et al concluded that hypocalcemia did not necessarily indicate a poor prognosis, and “thus the impact of hypocalcemia on the outcome seemed to be of relatively small importance.”^6^ We also concluded that there are more important circumstances influencing prognosis than hypocalcemia itself.

This study has some limitations. It was carried out in only 1 hospital, which may cast doubt on the generalizability of the results. Also, not all patients had the albumin value available, leading to the exclusion of 23% of the initial sample. However, the analysis included 638 patients, which is a greater sample size than the previous studies on hypocalcemia mentioned above.^6,17^ The retrospective nature of this study did not allow systematic data on the symptoms associated with hypocalcemia to be collected. The correction of total calcium for the albumin level is not a very accurate method for determining the real level of calcium, and the direct assessment of ionized calcium should be preferred.^23^ However, the available data did not allow the analysis based on ionized calcium, but the next step should be to carry out such an analysis.

## Conclusion

Hypocalcemia is associated with several types of cancer, but is more frequent in hematological, colorectal, lung, and thyroid cancers. A low calcium level measured at some point in a patient with cancer seems to not be significantly associated with lower survival. In this study, the normal corrected calcium was associated with a lower survival rate than low corrected calcium. However, corrected calcium is calculated for hypoalbuminemia, which has a significant influence on survival. Therefore, the influence of corrected calcium seems to be nonsignificant by itself. Other factors influencing survival were age and the primary cancer.

## Acknowledgments

This work was supported in part by the North Section of the Portuguese League against Cancer.

The authors would like to thank Mrs Ana Paula Costa for her administrative assistance and to Mr Luis Antunes for his advice with the statistical analysis.

## Conflicts of interest

The authors declare no conflicts of interest.
